# Effects of Steroid Hormones on Sex Differences in Cerebral Perfusion

**DOI:** 10.1371/journal.pone.0135827

**Published:** 2015-09-10

**Authors:** Carmen Ghisleni, Steffen Bollmann, Anna Biason-Lauber, Simon-Shlomo Poil, Daniel Brandeis, Ernst Martin, Lars Michels, Martin Hersberger, John Suckling, Peter Klaver, Ruth L. O'Gorman

**Affiliations:** 1 Center for MR-Research, University Children's Hospital Zurich, Zürich, Switzerland; 2 Zurich Center for Integrative Human Physiology, University of Zurich, Zürich, Switzerland; 3 Neuroscience Center Zurich, University of Zurich and ETH Zurich, Zürich, Switzerland; 4 Endocrinology, Department of Medicine, University of Fribourg, Fribourg, Switzerland; 5 Department of Child and Adolescent Psychiatry, University of Zurich, Zürich, Switzerland; 6 Department of Child and Adolescent Psychiatry and Psychotherapy, Central Institute of Mental Health, Medical Faculty Mannheim/ Heidelberg University, Mannheim, Germany; 7 Klinik für Neuroradiologie, University Hospital Zurich, Zürich, Switzerland; 8 Division of Clinical Chemistry and Biochemistry, University Children's Hospital Zurich, Switzerland; 9 Department of Psychiatry, University of Cambridge, Cambridge, England; 10 Department of Psychology, University of Zurich, Zürich, Switzerland; University of Pennsylvania, UNITED STATES

## Abstract

Sex differences in the brain appear to play an important role in the prevalence and progression of various neuropsychiatric disorders, but to date little is known about the cerebral mechanisms underlying these differences. One widely reported finding is that women demonstrate higher cerebral perfusion than men, but the underlying cause of this difference in perfusion is not known. This study investigated the putative role of steroid hormones such as oestradiol, testosterone, and dehydroepiandrosterone sulphate (DHEAS) as underlying factors influencing cerebral perfusion. We acquired arterial spin labelling perfusion images of 36 healthy adult subjects (16 men, 20 women). Analyses on average whole brain perfusion levels included a multiple regression analysis to test for the relative impact of each hormone on the global perfusion. Additionally, voxel-based analyses were performed to investigate the sex difference in regional perfusion as well as the correlations between local perfusion and serum oestradiol, testosterone, and DHEAS concentrations. Our results replicated the known sex difference in perfusion, with women showing significantly higher global and regional perfusion. For the global perfusion, DHEAS was the only significant predictor amongst the steroid hormones, showing a strong negative correlation with cerebral perfusion. The voxel-based analyses revealed modest sex-dependent correlations between local perfusion and testosterone, in addition to a strong modulatory effect of DHEAS in cortical, subcortical, and cerebellar regions. We conclude that DHEAS in particular may play an important role as an underlying factor driving the difference in cerebral perfusion between men and women.

## Introduction

Sex differences in the brain appear to play an important role in the prevalence and progression of various neuropsychiatric disorders, as well as in learning, emotion perception, and treatment response [1–3]. While early childhood disorders like autism and attention-deficit/hyperactivity disorder are more prevalent in males, anxiety and depression are more prevalent in females [1], and differences in the age of onset for schizophrenia have also been reported between men and women [4]. However, to date little is known about the cerebral mechanisms underlying these apparent differences, despite an increasing body of knowledge about differences in brain structure, function, and morphology between the sexes.

One of the most widely reported findings with regard to baseline brain physiology in men and women is that of an increased rate of perfusion or cerebral blood flow in women. Sex differences in cerebral perfusion have been observed using various techniques including single-photon emission computed tomography (SPECT), positron emission tomography (PET), Xenon-enhanced computed tomography, and arterial spin labelling (ASL) [5–9], both on a global level [5–7,9–11], and locally in posterior cingulate cortex, precuneus, and thalamus [8,12]. In addition to demonstrating higher perfusion during rest, women have also been reported to show higher perfusion during cognitive activity [2]. However, the underlying cause of these sex differences remains unclear and the factors modulating this sex difference in perfusion are poorly understood.

One contributing factor for the reported sex differences in perfusion may lie in the combinatory modulation of different steroid hormones (including sex hormones), since these hormones are known to influence the vascular response and to differ between men and women. Specifically, oestradiol, testosterone, and dehydroepiandrosterone sulphate (DHEAS) are thought to represent potential modulators of perfusion. Oestrogens enhance production or sensitivity to vasodilatory factors (for a review, see [13]), and have been shown to be positively related to cerebral blood flow (CBF) or perfusion in studies applying techniques such as Doppler ultrasound [14], SPECT [15], and PET [16]. Testosterone, on the other hand, exerts vasoconstrictive effects [13], and testosterone supplementation has been reported to decrease CBF in postmenopausal women [17]. In men, the local metabolism of testosterone into oestradiol via aromatase [18] might influence the relationship of circulating testosterone and perfusion to a significant degree. This mechanism may underlie the finding of an increase in CBF in hypogonadal men [19].

Sex differences are not only present in the sex steroids oestradiol and testosterone, but also in DHEAS, which is a precursor of sex steroids. Most studies reported higher levels of DHEAS in men than in women [20–23], while others reported no significant sex differences in DHEAS [24,25]. Nevertheless, a multitude of studies have shown the wide range of functions of DHEAS and its non-sulphated precursor DHEA (collectively referred to as "DHEA(S)") in human physiology, cardiovascular diseases, and brain function and diseases (for reviews, see [26–30]). A few studies reported positive associations between flow-mediated vasodilation of brachial artery and DHEA(S) in postmenopausal women [31,32], while others found no effect [33]. One study found a positive correlation between hippocampal perfusion measured with SPECT and DHEAS in patients with Alzheimer's disease but not in controls [34]. The role of DHEAS as an underlying factor in the sex difference in cerebral perfusion therefore remains unclear.

In this study, we investigate whether steroid hormone concentrations are linked to cerebral perfusion, and specifically whether hormone concentrations may explain the previously reported sex differences in perfusion. To our knowledge, no studies have yet investigated the relationships between serum sex hormones, DHEAS and cerebral perfusion in the same healthy volunteers. We use non-invasive ASL, which provides a quantitative measure of tissue perfusion, in contrast to the relative blood oxygenation level dependent response measured by functional magnetic resonance imaging (fMRI). The benefits and the wide range of possible applications of ASL have been demonstrated in a large body of studies in basic and clinical neuroscience (for a review, see [35]). Based on the known sex differences in perfusion and hormone levels, we hypothesized that perfusion correlates positively with oestradiol as both are higher in women but negatively with testosterone, which is lower in women. Although DHEAS is also lower in women, we hypothesized that it is positively related to perfusion as found in previous SPECT and ultrasound studies (see above).

## Methods

### Subjects

The subject group consisted of 44 adult volunteers (20 males), recruited by local advertisement. Subjects were excluded due to comorbid disorders affecting perfusion (*n* = 2), caffeine intake shortly before the measurement (*n* = 1), and technical problems with the ASL acquisition (*n* = 5). Perfusion data were acquired from the remaining 36 subjects (16 males) and entered into the analysis of the sex difference in perfusion (see Table 1 for demographics). Additionally, blood samples were collected for hormone assay (see below) for *n* = 35 subjects (15 males). Hematocrit (Hct) data were collected in a subset of *n* = 18 subjects (9 males). Subjects reported no history of neurological or psychiatric illness, illegal substance abuse, or use of psychotropic medication. All subjects refrained from caffeine, alcohol, and nicotine for 4 hours (2.5 hours for nicotine in one subject) before the experiment.

**Table 1:** Group demographics and hormone values for the voxel based analyses.

		Men				Women				
Analysis	Variable	*n* ^§^	Mean (*SD*)	Median	Range	*n* ^§^	Mean (*SD*)	Median	Range	*p* *
Sex difference in perfusion	Age [years]	16	33.7 (9.9)	32.4	21.4–50.6	20	30.3 (8.6)	27.3	21.0–48.4	.36^b^
Voxel-based correlation between perfusion and sex steroids	Age [years]	14	33.3 (10.5)	29.1	21.4–50.6	12	33.5 (9.7)	29.6	21.8–48.4	.96^a^
	Oestradiol [pmol/L]	14	99.73 (40.60)	91.75	59.38–203.90	12	332.70 (160.35)	331.30	103.10–587.80	< .001^a^
	Testosterone [nmol/L]	14	17.12 (4.72)	17.29	8.10–25.39	12	0.92 (0.45)	1.04	0.21–1.49	< .001^a^
Voxel-based correlation between perfusion and DHEAS	Age [years]	15	33.6 (10.2)	30	21.4–50.6	19	30.5 (8.8)	27.5	21.0–48.4	.49^b^
	DHEAS [μmol/L]	15	7.78 (4.34)	7.25	2.15–14.43	19	5.40 (2.55)	5.21	0.90–9.91	.07^a^

DHEAS, dehydroepiandrosterone sulphate. All median hormone values were within the reference range (for serum oestradiol in men: 93-276 pmol/L, in women: 110-2750 pmol/L; for serum testosterone in men: 7.6-31 nmol/L, in women: 0.2-1.8 nmol/L; for serum DHEAS in men: 1.2-13 μmol/L, in women: 1.0-9.2 μmol/L).

*p-value of comparison between men and women.

^a^ two-tailed t-test.

^b^ Wilcoxon rank sum test.

^§^ *n* differed for different analyses due to drop-outs (see Methods).

### Ethics Statement

The study was approved by the Ethics Committee of the Canton of Zurich, Switzerland. All subjects gave written informed consent.

### Hormone concentration acquisition

Serum concentrations of oestradiol, testosterone, and DHEAS were measured on an Elecsys 2010 using commercial Electro-Chemi-Luminescence Immuno-Assays (Roche Diagnostics, Rotkreuz, Switzerland) with coefficients of variations of 8.4% (256 pmol/L), 4.9% (7.4 nmol/L), and 4.7% (2.3 μmol/L), respectively.

Data were collected as part of a larger study investigating age-related cerebral changes of psychophysiological markers from childhood to adulthood. Perfusion data and blood samples were collected during the same measurement session. All measurements were performed in the afternoon or early evening. Hormone data from two female participants were incomplete (*n* = 1 female with missing oestradiol value, *n* = 1 female with missing DHEAS and oestradiol). Regression analyses between hormones and perfusion were performed both for the full group of subjects including all available hormone data and for a subset of participants excluding subjects taking hormonal contraceptives (*n* = 8 females) or medication affecting testosterone (*n* = 1 male). For all analyses, there was no difference in age between men and women (see Table 1).

### MRI-data acquisition

MR imaging studies were performed with a 3.0 T GE HD.xt whole-body MRI scanner (GE Healthcare, Milwaukee, WI, USA), using an 8-channel receive-only head coil and a body transmit coil. Cerebral perfusion images were collected during an eyes-closed resting condition with a background-suppressed, pulsed continuous arterial spin labelling (pCASL) sequence, using a 3D stack of spirals fast spin echo readout [36]. Thirty-two axial slices were collected with a repetition time of 5.5 s and an echo time of 25 ms, a slice thickness of 4 mm, a field of view of 24 cm, 3 Nex, a nominal in-plane resolution of 1.9 × 1.9 mm^2^, and a total scan time of 5 min 17 s. A post-labelling delay of 1.5 s was used to reduce errors from transit time effects [37]. Structural images were obtained with a 3D T1-weighted gradient echo pulse sequence (number of slices = 172, slice thickness = 1.0 mm, repetition time = 9.94 ms, echo time = 2.948 ms, inversion time = 600 ms, field of view = 256 mm × 192 mm, flip angle = 8°, matrix = 256 × 192, reconstructed voxel resolution: 1 × 1 × 1 mm). The participants were provided with earplugs.

### MRI preprocessing

**Fig 1:**
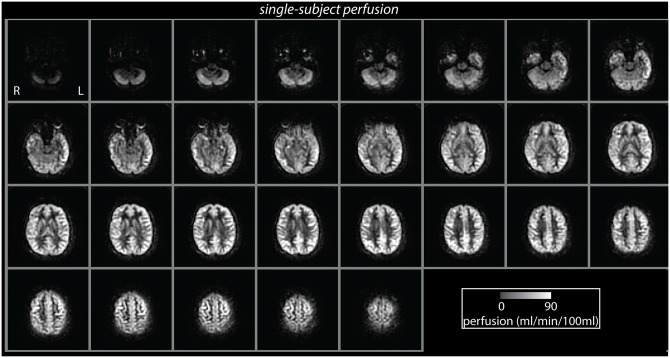
Resting perfusion map acquired from a single participant, shown in radiological orientation (scale: 0–90 ml/min/100ml).

The perfusion images were quantified using the model proposed by Alsop and Detre [37], with additional terms included to represent the finite labelling duration [38] and to correct for incomplete recovery of the magnetisation in the reference image due to the saturation applied t_sat_ (2,000 ms) before imaging. The perfusion was calculated according to the following equation [37,39]:

$$f=\frac{\lambda }{2\alpha T_{1b}\left(1-\;e^{-\frac{\tau }{T_{1b}}}\right)}\;\frac{\left(S_{ctrl}-\;S_{lbl}\right)\left(1-\;e^{-\frac{t_{sat}}{T_{1g}}}\right)}{S_{ref}}\;e^{\frac{w}{T_{1b}}}$$ (1)

where f is the perfusion (in ml/min/100 ml), S_ctrl_ − S_lbl_ is the difference image (control-label), and S_ref_ is a proton-density weighted reference image. λ is the blood brain partition coefficient (0.9), α is the inversion efficiency, T_1b_ is the T_1_ of blood (1600 ms), *T*_*1g*_ is the T_1_ of grey matter (1200 ms), w is the post-labelling delay (1.5 s), and τ is the labelling duration (1.5 s). The labelling efficiency is given by the product of the pCASL labelling efficiency (0.95) and an additional efficiency factor, which incorporates the loss of efficiency from the background suppression (0.75). This equation includes an additional term to correct for incomplete recovery of the magnetisation in the reference image due to a saturation pulse applied t_sat_ (2,000 ms) before imaging [39]. The model assumes that the labelled spins remain primarily in the microvasculature rather than exchanging with tissue water, so the T_1_ of blood is used for quantification [38,39]. A representative perfusion map from one participant is shown in Fig 1.

The perfusion images for each subject were normalised to a custom perfusion template in the Montreal Neurological Institute space using the flirt algorithm in FSL (fsl.fmrib.ox.ac.uk/fsl) with the correlation ratio as the cost function. For later statistical analyses, a study-specific template was generated from the normalised perfusion images of 35 healthy adult subjects by first concatenating the images in time and subsequently calculating a mean image across time using the fslmerge and fslmaths utilities from FSL. Finally, the Brain Extraction Tool from FSL was applied to mask this mean image.

### Statistical analyses

Statistical analyses of the non-imaging data were performed using MATLAB and Statistics Toolbox Release 2012b (The MathWorks, Inc., Natick, Massachusetts, United States), and IBM SPSS Statistics, Version 20. In cases of non-normally distributed data (tested with Lilliefors test), a non-parametric Wilcoxon rank sum test was used (see Table 1).

Whole brain perfusion values were extracted to examine differences in global perfusion between men and women and to examine the effects of each hormone on the whole brain perfusion. Specifically, a whole brain mask was derived from the AAL atlas [40] and registered to the CASL images for each subject in native space, using the flirt algorithm in FSL (fsl.fmrib.ox.ac.uk/fsl), with the correlation ratio as the cost function. The individual subject's perfusion image was then masked with this image using fslmaths, and the mean perfusion signal from this masked image was calculated with fslstats (fsl.fmrib.ox.ac.uk/fsl). A multiple regression model in SPSS (Enter method) was then used to test for the differential effect of each hormone on the perfusion, with whole brain perfusion as the dependent variable and age, oestradiol, testosterone, and DHEAS levels as independent variables.

In all voxel-based analyses on perfusion data, age was included as a covariate or regressor due to the known effect of age on perfusion [e.g. 8]. Both the sex difference and the correlations between perfusion and hormone levels were tested using the nonparametric permutation-based methods implemented in the Cambridge Brain Analysis (CamBA) software [41,42]. The following general linear model was used for all analyses:

$$P\;=\;a_{0}+a_{1}\text{ independent variable }+a_{2}\text{ age }+e$$ (2)

where *P* is the perfusion at a particular voxel, *a*_*0*_ is the mean effect across all subjects, *a*_*1*_ is the coefficient relating the independent variable vector to perfusion at a particular voxel (i.e. sex in the sex difference analysis and hormone values in the correlation analyses), *a*_*2*_ is the coefficient for the covariate vector of age, and *e* is an error term.

This model was regressed at each intra-cerebral voxel onto the observed data to yield a test statistic map of a non-parametric *t*-value given by the coefficient *a*_*1*_ divided by its standard error. The model was also regressed 32 times at each voxel after random permutations of the vector coding the respective independent variable (i.e. sex or hormone values) within the subject groups, thus breaking the association between the individual subjects and their individual sex or hormone levels (see Table 1 for composition of subject groups). The resulting permutation distributions of *a*_*1*_, combined over all voxels, were used to derive a preliminary, voxel-level threshold at *p* = .05, which was then applied to observed and permuted maps identically. The sum or "mass" of the resulting suprathreshold voxel statistics was computed for each cluster in both the observed and permuted maps, and these values were ordered to sample the permutation distribution under the null hypothesis of zero difference in perfusion between the sexes or zero correlation between perfusion and hormones within the groups. The mass of each cluster in the observed map was then tested against the critical values obtained from the corresponding permutation distribution. The significance thresholds were corrected for multiple comparisons by setting the number of error clusters accepted to < 1 per image. For the permutation data acquired in the present study, this threshold corresponds to a familywise error (FWE)-corrected p-value of *p* < 0.004.

To determine the degree to which the observed voxel-based hormone correlations may contribute to the observed sex difference, the number of overlapping significant voxels between the sex difference map and the hormone correlation maps was calculated and expressed as a percentage of the voxels in the sex difference map.

In order to assess the impact of sex-based differences in brain structure on the perfusion results, voxel based morphometric [43,44] differences between men and women were tested with the standard DARTEL pipeline using SPM8 (Wellcome Trust Centre for Neuroimaging, London, UK; www.fil.ion.ucl.ac.uk/spm). T1-weighted images from each participant were first segmented into grey, white, and cerebrospinal fluid images [45]. Segmented grey matter (GM) images were “modulated” using non-linear warping procedures that correct for global brain differences and align homologous brain regions into a common space [43,46]. The resulting images were then smoothed using a Gaussian kernel with 8 mm full-width at half maximum [43]. Between-group analysis was performed on the smoothed images to determine brain voxels where local GM density and volume differed between males and females, after controlling for differences in brain size (using total intracranial volume measures as covariate).

## Results

The results of the hormone assays are given in Table 1. As to be expected, oestradiol levels were higher in women (*M* = 332.7 pmol/L, *SD* = 160.35) than in men (*M* = 99.73 pmol/L, *SD* = 40.6, *p* < .001), testosterone levels were higher in men (*M* = 17.12 nmol/L, *SD* = 4.72) than in women (*M* = 0.92 nmol/L, *SD* = 0.45, *p* < .001), and DHEAS levels were higher in men (*M* = 7.78 μmol/L, *SD* = 4.34) than in women (*M* = 5.4 μmol/L, *SD* = 2.55) on a trend level (*p* = .07). Hematocrit values were higher in men (*M* = 0.45, *SD* = 0.02) than in women (*M* = 0.40, *SD* = 0.02, *p* < .001).

**Fig 2:**
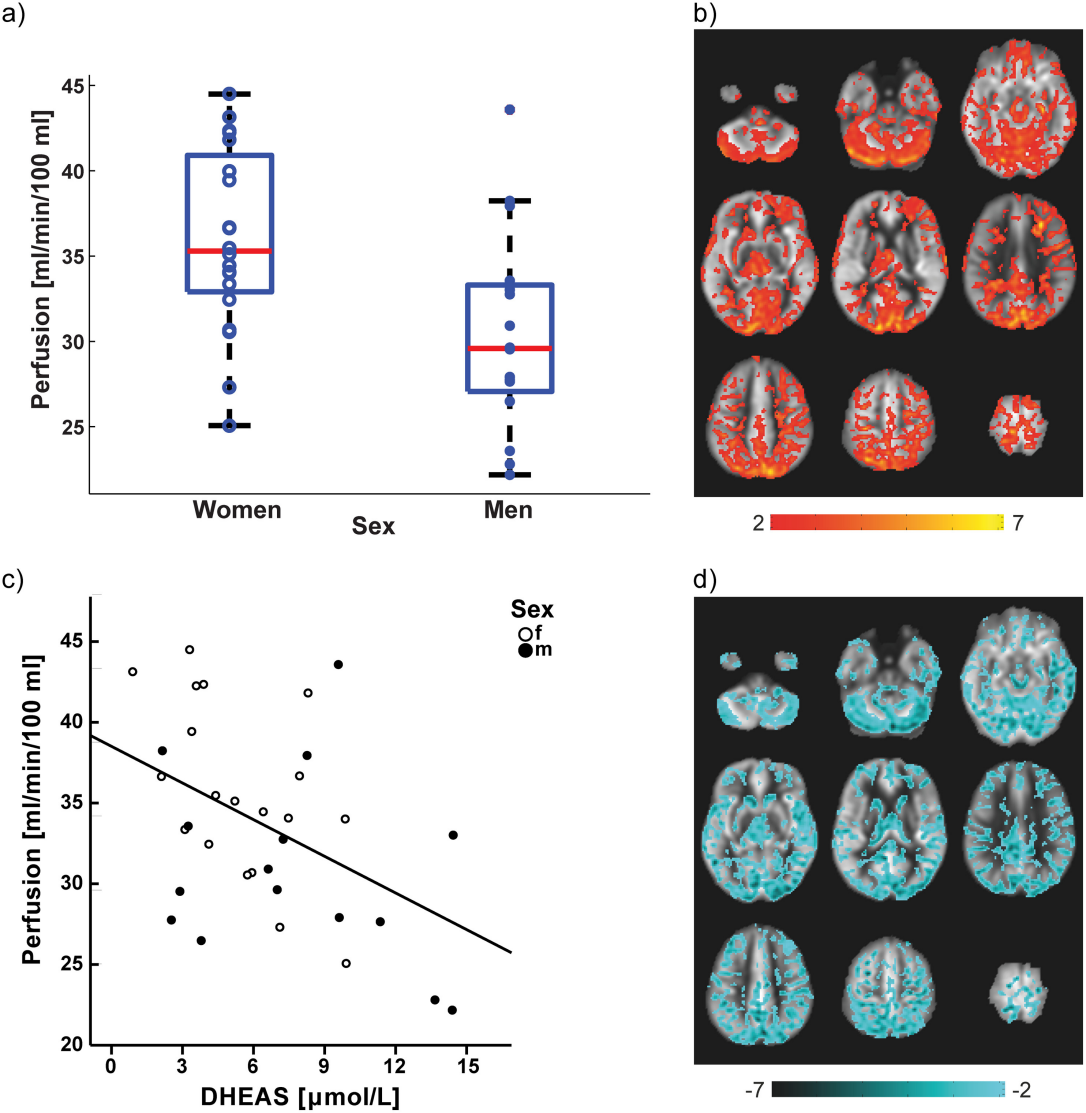
Women show higher perfusion than men and DHEAS correlates negatively with perfusion. a) Sex difference in whole brain grey matter perfusion: perfusion is higher in women (*M* = 35.97 ml/min/100 ml, *SD* = 5.37) than in men (*M* = 30.47 ml/min/100 ml, *SD* = 5.91, *p* = .006). Single dots represent the subjects' individual values. The horizontal line within the boxes indicate medians, the edges of the boxes are the 25^th^ and 75^th^ percentiles, and the whiskers represent 1.5 times the interquartile range. b) Sex difference (women > men) in regional perfusion: women show higher regional perfusion than men (*p* = .004, FWE-corrected). c) Simple regression analysis with whole brain perfusion values as the dependent variable and DHEAS as the only predictor: a significant model was found (*p* = .007, adjusted *R* ^*2*^ = .180) with a standardised β = -.452 for DHEAS. d) DHEAS effects in men and women: DHEAS correlates negatively with regional perfusion in both sexes (*p* = .004, FWE-corrected). Colour bar in a) and c) denotes a non-parametric *t* score, given by *a1*/[standard error(*a1*)], see methods. Images are shown in neurological orientation. Slices are at MNI z-coordinates -45, -30, -15, 0, 15, 30, 45, 60, 75 (from top left to bottom right).

As expected from previous reports, both the voxel-based perfusion analysis and the global perfusion analysis demonstrated that perfusion is higher in women. Across the whole brain, perfusion was higher in women (*M* = 35.97 ml/min/100 ml, *SD* = 5.37) than in men (*M* = 30.47 ml/min/100 ml, *SD* = 5.91, *p* = .006, see Fig 2a). The voxel-based analysis of the sex difference in perfusion revealed an extensive cluster in which women showed higher regional perfusion than men (*p* = .004, FWE-corrected). This cluster included frontal, parietal, temporal, and occipital regions as well as thalamus, basal ganglia, and cerebellum (see Fig 2b). There were no regions in which perfusion was significantly higher in men than in women in the voxel-based analysis.

The multiple regression analysis of the whole brain perfusion data with age, oestradiol, testosterone, and DHEAS levels as predictors revealed a significant model (*p* = .047, R = .532, adjusted *R* ^*2*^ = .181), of which DHEAS emerged as the only significant predictor (standardised β = -.536, *p* = .020). The standardised β and *p*-values for the other predictors are as follows: age (standardised β = -.273, *p* = .216), oestradiol (standardised β = -.009, *p* = .961), and testosterone (standardised β = -.134, *p* = .530). Thus, the regression analysis was repeated with only DHEAS as a predictor, resulting in a significant model (*p* = .007, adjusted *R* ^*2*^ = .180) and a standardised β = -.452 for DHEAS (see Fig 2c). The regression with DHEAS as the only predictor in the subgroup of subjects excluding participants on hormonal contraception or medications affecting testosterone revealed a significant model (*p* = .041, adjusted *R* ^*2*^ = .128) and a standardised β = -.404 for DHEAS. An additional single regression analysis for oestradiol as the only predictor did not reach significance (*p* = .897, standardised β = .023). A single regression analysis for testosterone revealed a significant model (*p* = .032, standardised β = -.363). After repeating the regression between perfusion and testosterone for men and women separately, no significant model was observed for men (*p* = .476, standardised β = 0.200), but testosterone was negatively correlated with perfusion in women (*p* = .025, standardised β = -.498).

In the subset of *n* = 18 participants with available hematocrit (Hct) data, an additional multiple regression was performed including Hct in addition to DHEAS, age, oestradiol, and testosterone. In this smaller sub-sample the model was no longer significant (*p* = .50, Adjusted *R* ^*2*^ = -0.021), but DHEAS remained the strongest predictor of whole brain perfusion (standardised β = -.679, *p* = 0.08) compared to Hct (standardised β = -.205, *p* = 0.59), age (standardised β = -.388, *p* = 0.31), oestradiol (standardised β = .003, *p* = 0.99), or testosterone (standardised β = .183, *p* = 0.64). The regression was repeated after correcting the perfusion values for differences in the T1 of blood relative to the value of 1600 ms assumed in the quantification (see Eq (1) above), using the relationship between Hct and the blood T1 reported in [47]. This regression with Hct-corrected perfusion values showed similar results with DHEAS emerging as the strongest predictor of perfusion (standardised β = -.718, *p* = 0.07) compared to Hct (standardised β = 0.005, *p* = 0.99), age (standardised β = -.411, *p* = .288), oestradiol (standardised β = .002, *p* = 0.99), or testosterone (standardised β = .192, *p* = 0.63).

The voxel-based correlation analysis between cerebral perfusion and DHEAS levels revealed an extensive cluster showing a negative correlation (*p* = .004, FWE-corrected). This cluster largely overlapped with the observed sex difference cluster and thus also included frontal, parietal, temporal, and occipital regions as well as thalamus, basal ganglia, and cerebellum (see Fig 2d). The calculated overlap between the sex difference map and the DHEAS correlation map was 65%. This analysis was rerun in two analyses which contained only subjects of one sex and the same DHEAS effect was seen in both men and women separately. Keeping the significance level correction to less than one error cluster per image, only the correlation analysis between testosterone and cerebral perfusion in women revealed two small and dispersed negative clusters (*p* = .003, FWE-corrected). These clusters included bilateral primary and secondary visual cortex extending into precuneus as well as in bilateral thalamus extending into left ventral temporal and cerebellar regions. The ratio between the overlapping significant voxels in both the sex difference and this testosterone correlation map to the whole number of significant voxels in the sex difference map was only 3%. All other voxel-based correlation analyses (i.e. oestradiol in women, oestradiol in men, and testosterone in men) did not yield any significant clusters at the chosen significance level (with less than one error cluster per image). At a family-wise error-corrected significance level of *p* < .05, no significant GM differences between men and women were observed.

## Discussion

This study provides the first investigation of the link between steroid hormone levels and perfusion on both a global and regional level. In both the whole brain perfusion analysis and the voxel-based analysis our results replicated the well-known finding that women show higher perfusion than men [5,6,8,12]. Moreover, we demonstrate for the first time that DHEAS was the only significant predictor of whole brain perfusion amongst the steroid hormones investigated in this study. Additionally, DHEAS showed a strong and widespread negative correlation with cerebral perfusion in the voxel-based analysis, and the corresponding correlation map also overlapped to a large degree (65%) with the voxel-based sex difference map. While the direct implications of this apparent link between DHEAS and perfusion remain unclear, the higher perfusion in women may partially explain the difference in treatment response observed between men and women [2]. Investigating the biochemical basis of the reported sex differences and the link between hormones and cerebral perfusion may therefore lend important insight into the neurobiological basis of various neuropsychiatric disorders, and brain recovery processes.

Our finding of a negative relationship between DHEAS and cerebral perfusion is contrary to our hypothesis, but consistent with studies showing higher DHEAS levels in men [20–23], coupled with the known higher perfusion in women [5,6,8,9,12]. Additionally, the sex difference in DHEAS is most significant starting around puberty [21,48,49], and sex differences in cerebral perfusion have been reported to become significant only in boys and girls older than 12 years of age [12]. Broadly, these results suggest that DHEAS may play a strong modulatory role in explaining the sex difference in cerebral perfusion reported previously.

Sex differences have also been reported in cerebral autoregulation in healthy subjects [50]. The effects of DHEAS on endothelial function, blood flow [28], and its relationship with cerebral perfusion found in this study may point to a role for DHEAS in cerebral autoregulation. While the precise mechanism underlying the regulation of cerebral perfusion is not yet fully understood, DHEAS may play a differential role in cerebral autoregulation in men and women, for example due to genetic influences varying between the sexes (see discussion in [23]). Future studies examining both vascular flow and perfusion following DHEAS supplementation may be able to elucidate further the role of DHEAS in blood flow regulation in men and women.

Interestingly, in postmenopausal women, Akishita et al. [32] also observed DHEAS as the only significant predictor of flow-mediated vasodilation in several multiple regression models including plasma oestradiol, testosterone, and cortisol levels as well as age and coronary risk factors as predictors. However, in their study DHEAS was positively associated with flow-mediated vasodilation. While our finding of a negative relationship of DHEAS and perfusion supports a role of DHEAS in the sex difference in cerebral perfusion, it also seems to contradict the more frequently reported beneficial effects of DHEAS on a different parameters related to blood flow. The reason for these seemingly contradictory results may lie in the differences in the parameters and/or subject populations investigated and in the different methods applied in other studies. For example, a peripheral measure of blood flow in the arm of postmenopausal women was used in other studies, of which some found a positive association between DHEAS and blood flow [31], while others did not [33]. In contrast to these non-cerebral and less direct measurements of perfusion, Murialdo et al. [34] reported a positive correlation between DHEAS levels and hippocampal SPECT findings in patients with Alzheimer's disease but not in controls. Since DHEA(S) has been shown to be involved in numerous physiological functions including endothelial function and blood flow but also including body composition, insulin sensitivity, and cardiovascular disease risk [28], the effects of DHEA(S) might present differently in patient groups or older subjects compared to healthy and younger subjects. Maninger et al. [27] discussed the scarcity of evidence for beneficial effects of DHEA(S) treatment in healthy subjects and argued that benefit from such treatments may be more likely observed in medically or neuropsychiatrically ill patients. Thus, while our results may seem surprising in the light of previous literature, the mechanisms of action of DHEA(S) may differ depending on the subject group investigated, and results may depend on the investigational methods. The precise underlying mechanisms and their differences remain unclear and warrant further study. However, our observation of a negative relationship between DHEAS and cerebral perfusion is consistent with the higher perfusion observed in women and the higher DHEAS levels typically observed in men.

Our other hypotheses regarding the association between cerebral perfusion and oestradiol and testosterone were partly confirmed. As hypothesised, cerebral perfusion correlated negatively with testosterone in women. In men, however, no significant correlation between perfusion and testosterone was found. Although more modest, the negative correlation of testosterone with global and local perfusion in women suggests a partial role of this hormone in accounting for sex differences in cerebral perfusion, at least in women. The results of the sex steroid analyses in men, however, do not imply a strong modulatory role of testosterone on cerebral perfusion or a predominant role as an underlying factor for the sex differences in perfusion. Oestradiol also does not appear to have a strong modulatory role on cerebral perfusion, which was surprising and contrary to our hypothesis. Oestrogens have been hypothesised to underlie the increased rates of cerebral glucose and oxygen metabolism seen in women, which in turn have been suggested to underlie the sex difference in perfusion [11,51], under the assumption that a higher perfusion is required to support a higher level of metabolism. In the present study neither oestradiol nor testosterone exerted a large effect on global perfusion, but these results might also reflect the absence of a direct association between circulating levels of testosterone and oestradiol and their local concentration in the brain [18]. Thus, apart from DHEAS, the circulating levels of steroid hormones investigated in this study seem less likely to represent direct or primary modulators of perfusion and major factors underlying the sex difference in cerebral perfusion.

One factor not considered in the present study was the effect of sex hormone binding globulin (SHBG), a glycoprotein which plays and important role in modulating the amount of free oestradiol and testosterone. SHBG has been related to psychopathology in patients with eating disorders [52], who have also been reported to show reduced perfusion [53]. Since only a small fraction of 1–2% of oestradiol and testosterone are free and unbound, SHBG binding (and to a lesser extent serum albumin binding) is an important factor determining the bioavailable fraction of these steroids and hence their uptake into the brain. In healthy participants, the unbound and bound fractions of oestradiol and testosterone are in equilibrium, keeping the concentration of the free (active) hormone relatively constant within the normal range. However, in future studies it would be interesting to examine individual differences in perfusion in the context of SHBG and free (active) oestradiol and testosterone levels since these factors may be more directly related to the cerebral concentrations of oestradiol and testosterone. DHEAS is primarily bound to serum albumin, an abundant protein which shows a lower affinity and a shorter dissociation time for steroid hormones relative to SHBG [54]. This shorter dissociation time is thought to account for the increased rate of transport of albumin-bound steroids through the blood brain barrier relative to globulin-bound hormones, as the shorter dissociation time (probably on the order of milliseconds) for albumin-bound steroids is short relative to the capillary transit time [54]. The stronger association observed between DHEAS and perfusion, relative to that between oestradiol and testosterone and perfusion, may therefore reflect a more direct association between circulating and cerebral concentrations of DHEAS relative to that of the sex steroids, but further studies investigating SHBG and free concentrations of oestradiol and testosterone would be needed to confirm this hypothesis.

In addition, circulating levels of oestradiol, testosterone, and DHEAS may not completely reflect their concentrations in the brain because these steroid hormones are not only secreted by endocrine glands (i.e. ovaries, testes, adrenals), but also synthesised de novo in neuronal tissue from their respective precursor hormones. It has been estimated that this local synthesis in peripheral tissue from inactive adrenal precursors might be as high as 30 to 50% for the total androgens in men and up to 75% for oestrogens in premenopausal women [55]. Thus, the intracellular levels in the brain may not translate into parallel changes in circulating levels of these hormones [56]. Labrie et al. [56] suggested that future studies should additionally investigate the levels of the derivates of the hormones of interest since these might be the most reliable estimate of the total androgen pool. Future studies in healthy human subjects additionally investigating these derivates in combination with other known vasomodulatory factors like nitric oxide [13,57,58] may help to elucidate further the modulatory influence of the different steroid hormones on perfusion and their role as factors explaining the sex difference in cerebral perfusion.

### Limitations

Given the higher perfusion values in GM relative to white matter, structural differences in brain volume between men and women could confound the assessment of perfusion differences. However, in our sample no significant differences in GM volume were observed with a corrected significance threshold. Therefore, the results of the present study are unlikely to be driven by differences in GM between men and women.

Hematocrit differences present another potential confound in the analysis of sex differences in perfusion between men and women, since the longitudinal relaxation time T1 of blood is related to Hct [47], and the blood T1 is an important parameter in the quantification of perfusion (see Eq (1) above). In the present study, Hct data were only available for a subset of 18 participants, but in this subgroup DHEAS remained the strongest predictor of perfusion both before and after correcting the whole brain perfusion values for Hct differences. However, the sex difference in perfusion between men and women was no longer significant in this smaller subsample, either correcting for Hct effects or using the uncorrected perfusion values, probably due to the lower statistical power and smaller sample size. In the full sample, assuming literature values for the Hct in men (0.42) and women (0.40) for the missing Hct values, the Hct-corrected whole brain perfusion remains significantly higher in women (*M* = 33.3 ml/min/100 ml, *SD* = 4.9) than in men (*M* = 29.1 ml/min/100 ml, *SD* = 6.0, *p* = .03). Therefore, the sex difference in whole brain perfusion between men and women appears to be at least partially independent from hematocrit, but the extent to which Hct affects apparent perfusion differences between men and women should be explored in a larger sample.

DHEAS was selected as a measurement target in preference to DHEA on the basis of its diurnal stability and longer half-life [28,59] and availability at our institution, but the effects of DHEA on perfusion would also be interesting to measure, since it crosses the blood brain barrier directly. Additionally, we did not measure progesterone levels, so it is not known to what extent progesterone affects cerebral perfusion. Female participants were asked for the date of their last period, but due to the variability in cycle length and ovulation time, it was not possible to confirm the menstrual cycle phase accurately. Future studies examining serum progesterone as well as oestradiol, testosterone, and DHEAS levels, with optimal control for menstrual cycle effects would be needed to further corroborate the findings of this study.

## Conclusion

This study has replicated the well known sex difference in cerebral perfusion, with women showing significantly higher global perfusion. Moreover, the correlation analyses between perfusion and the steroid hormones revealed a strong modulatory effect of DHEAS on perfusion, with modest sex-dependent correlations with testosterone. These results demonstrate for the first time that steroid hormones contribute to the observed sex difference in perfusion, and that DHEAS in particular may play an important role as an underlying factor accounting for the sex difference in cerebral perfusion.
